# H19 lncRNA in programming prenatal development and DOHaD due to maternal obesity

**DOI:** 10.1016/j.lfs.2026.124360

**Published:** 2026-03-27

**Authors:** Sharmeen Islam, Min Du

**Affiliations:** a Nutrigenomics and Growth Biology Laboratory, Department of Animal Sciences, Washington State University, Pullman, WA, 99164, USA

**Keywords:** H19, Maternal obesity, GDM, Offspring, miRNA, Development

## Abstract

Maternal obesity is a major global health challenge worldwide, significantly increasing the risk of pregnancy complications and long-term metabolic disorders in offspring. Maternal obesity, including associated gestational diabetes, induces epigenetic modifications that can reprogram fetal development and predispose children to lifelong health issues. Long non-coding RNA (lncRNA) H19, one of the first and most extensively studied lncRNAs, plays a pivotal role in developmental programming by regulating gene imprinting, microRNA processing, protein stabilization, and signaling pathways critical for growth and metabolism. Dysregulation of H19 expression under maternal obesity alters the H19/*Igf2* (insulin-like growth factor 2) imprinting axis, disrupts skeletal muscle development, and modifies osteogenic and neurogenic pathways, thereby contributing to systemic insulin resistance, metabolic dysfunction, and neuro-disorder in offspring. This review highlights how maternal obesity reprograms offspring health through H19-mediated epigenetic and post-transcriptional regulation, emphasizing its role in the developmental origins of health and disease framework. Understanding the role of H19 offers valuable opportunities to develop targeted interventions that may reduce the transgenerational effects of obesity.

## 1. Introduction

### 1.1. Impact of maternal obesity and gestational diabetes on offspring health

Obesity is a global epidemic that disproportionately affects women of childbearing age, with more than 40% of American women entering pregnancy obese [1]. Maternal obesity (MO) increases the risk of pregnancy-related complications, including gestational diabetes mellitus (GDM), preeclampsia, congenital malformations, and abnormal fetal growth, while also predisposing offspring to obesity, type 2 diabetes (T2D), and cardiovascular disease later in life [2]. MO creates an adverse intrauterine environment that leads to permanent structural and functional changes in fetal tissues. One critical mechanism underlying is epigenetic programming, where maternal metabolic status reshapes gene expression patterns that persist into adulthood [3].

Gestational diabetes (GDM) is a group of metabolic disorders characterized by impaired carbohydrate metabolism, where glucose is underutilized as an energy source and excessively produced through inappropriate gluconeogenesis and glycogenolysis, leading to hyperglycemia during pregnancy [4]. GDM develops when pancreatic insulin secretion fails to adequately compensate for the physiological insulin resistance that occurs during pregnancy [5]. In the United States, GDM affects approximately 2–10% of pregnancies annually, and its prevalence has risen in parallel with increasing rates of obesity [6]. The likelihood of developing GDM is approximately two-, four-, and eightfold higher in overweight, obese, and severely obese women, respectively, compared with women of normal pre-pregnancy weight [7]. Consistent with this, epidemiological studies demonstrate a strong association between MO and increased GDM risk [8]. Women with GDM are at significantly greater risk of developing type 2 diabetes mellitus and impaired insulin signaling later in life [9].

Beyond maternal health, accumulating evidence suggests that alterations in the maternal metabolic environment due to GDM and/or MO during critical windows of fetal development can have lasting consequences on offspring health [10]. Infants born to women with GDM are at an increased risk of perinatal complications and tend to have higher birth weights primarily due to increased fat mass rather than lean mass accretion [7,11]. The intrauterine environment in GDM adversely affects fetal metabolic programming and predisposes offspring to lifelong overweight and metabolic disorders, including obesity and diabetes [12].

The long-term consequences of MO and GDM on offspring health are increasingly attributed to developmental reprogramming driven by epigenetic modifications. Among these mechanisms, imprinted genes – key regulators of fetal growth and metabolic tissue development - are particularly vulnerable to disruptions in the intrauterine environment. Altered regulation of these loci provides a mechanistic bridge between maternal metabolic dysfunction and persistent disease susceptibility in offspring [3]. Notably, the imprinted long non-coding RNA (lncRNA) H19 has emerged as a critical mediator of this process, given its central role in coordinating fetal growth and metabolic tissue development [3,13].

## 2. Long non-coding RNA H19

### 2.1. Structure of H19

**Fig. 1.**
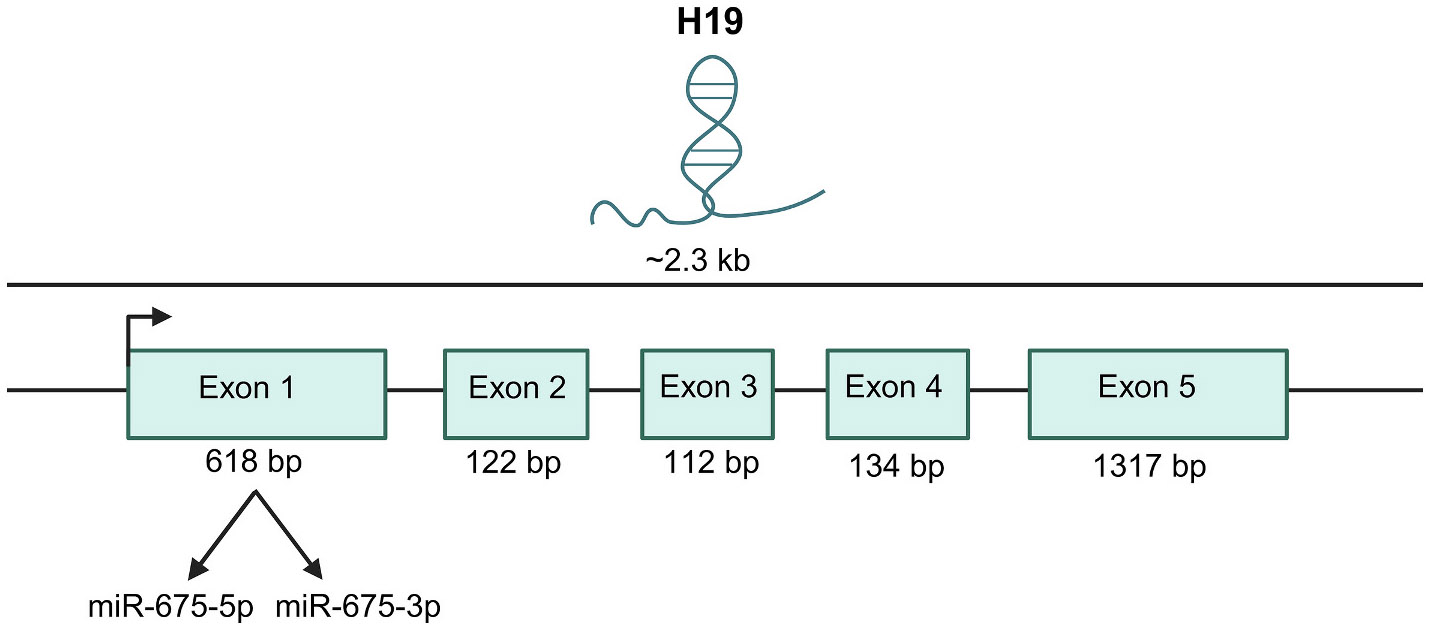
Structure of H19. Illustrating the structure of H19, including its exons and the embedded microRNAs miR-675-5p and miR-675-3p.

H19 was the first long non-coding RNA to be discovered and remains one of the most extensively studied lncRNAs [14]. In humans, H19 is located on chromosome 11, while in mice it resides on chromosome 7. Both human and mouse H19 consist of five exons and four introns, producing a ~ 2.3 kb spliced transcript that localizes to both the nucleus and cytoplasm and contains a 5′ cap and 3′ poly-A tail (Fig. 1) [15,16]. Comparative analyses across multiple species, including human, mouse, cat, and rabbit, reveal a highly conserved secondary structure, indicating that H19 functions as a structured regulatory RNA rather than a protein-coding transcript [17]. Consistent with this, its tightly regulated, stage and tissue specific expression further supports a functional role for H19 during development [18].

In addition to H19 lncRNA, the H19 locus generates several other transcripts. The first exon of H19 produces the conserved microRNAs miR-675-5p and miR-675-3p (Fig. 1) [13]. This locus also gives rise to two antisense transcripts, 91H and H19 Opposite Tumor Suppressor (HOTS) [19,20]. The human 91H transcript (~120 kb) spans the imprinting control region (ICR), the entire H19 gene, and downstream enhancers regulating both H19 and *Igf2* expression [19]. The HOTS transcript (~6 kb) is polyadenylated, contains a CpG island promoter, extends upstream and downstream of H19, is conserved in primates but not mice, and, like H19, is maternally expressed, encoding a nuclear-localized protein implicated in gene regulation [20].

### 2.2. Imprinting mechanism of H19

**Fig. 2.**
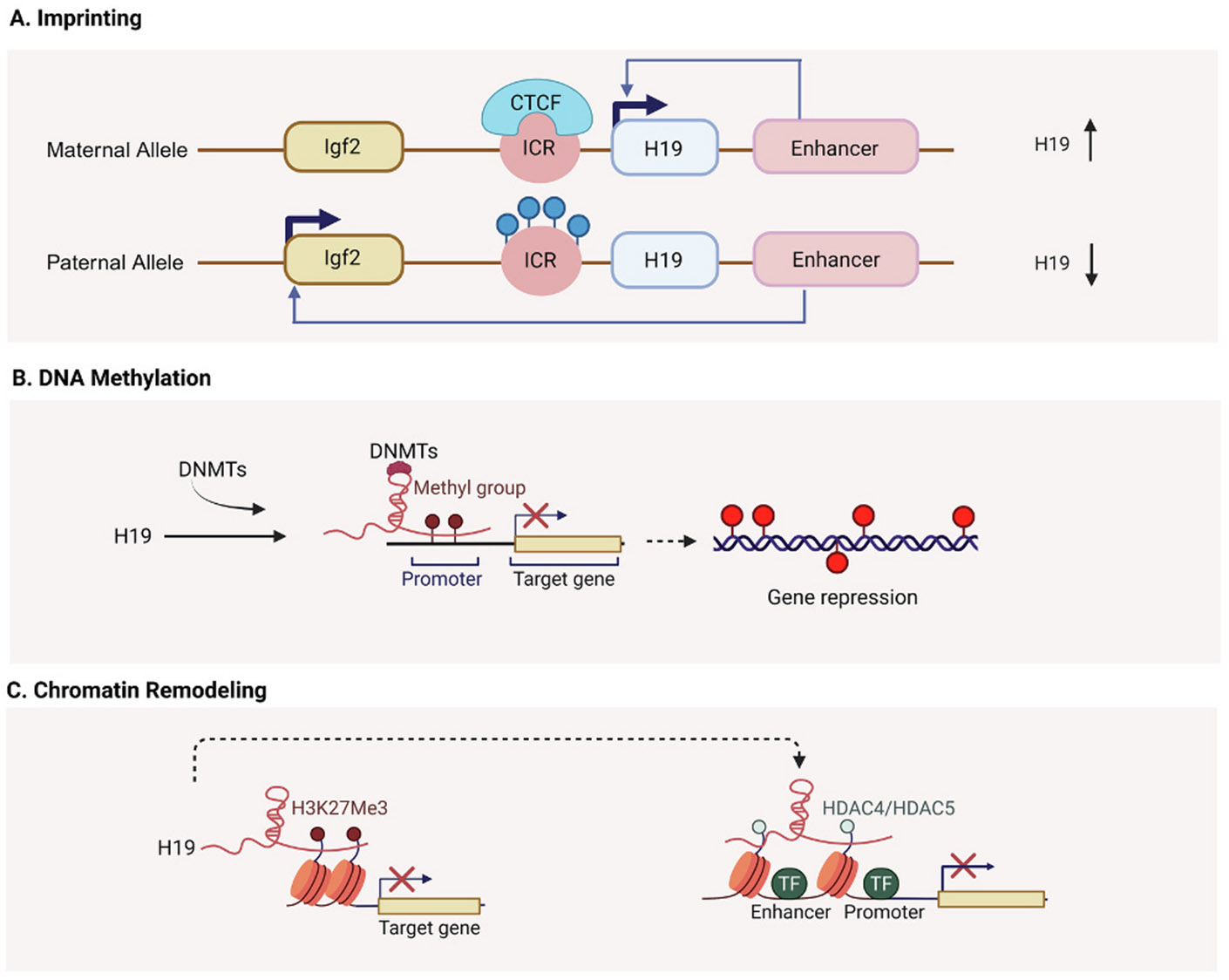
Epigenetic regulation of H19. (A) Allele-specific imprinting at the H19/*Igf2* locus mediated by differential ICR methylation and CTCF binding, resulting in maternal H19 expression and paternal *Igf2* expression. (B) H19-mediated recruitment of DNA methyltransferases (DNMTs) to target promoters, leading to DNA methylation and gene repression. (C) H19-driven chromatin remodeling through interaction with histone modifiers, including deposition of H3K27me3 or recruitment of HDAC4/5, thereby suppressing enhancer–promoter activity and transcriptional output.

H19 is part of an imprinted gene network (IGN) that plays an essential role in regulating embryonic growth [21]. It is located adjacent to the growth-promoting gene *Igf2,* and due to genomic imprinting, H19 is expressed from the maternal allele while *Igf2* is expressed from the paternal allele [13]. This parent-of-origin–specific expression is controlled by two key cis-regulatory elements: a set of shared enhancers downstream of the H19 gene and ICR positioned between H19 and *Igf2* (Fig. 2) [13]. The ICR contains four binding sites for the insulator protein CTCF and exhibits allele-specific DNA methylation patterns [22]. On the maternal allele, the ICR is hypomethylated, allowing CTCF binding, which blocks enhancer interaction with the *Igf2* promoter while facilitating enhancer access to the H19 promoter, thereby promoting H19 expression and silencing *Igf2* (Fig. 2) [22]. In contrast, on the paternal allele, the ICR is hypermethylated, preventing CTCF binding and enabling the enhancers to activate *Igf2* transcription while repressing H19 expression (Fig. 2) [23]. Disruption of imprinting at the H19/*Igf2* locus is associated with growth disorders, where maternal ICR hypermethylation leads to *Igf2* overexpression and Beckwith–Wiedemann syndrome, whereas paternal ICR hypomethylation reduces *Igf2* expression and contributes to growth restriction in Silver–Russell syndrome [24].

### 2.3. MO inducing H19 expression

In the fetuses of obese mothers, ICR that regulates H19 and *Igf2* expression becomes dysregulated on the paternal allele. In MO, alterations in the ICR lead to loss of imprinting, resulting in biallelic H19 expression and consequently elevated H19 levels [13]. This increase in H19 is accompanied by reduced *Igf2* expression in skeletal muscle tissue [13]. Elevated fetal H19 expression negatively affects myogenic cell behavior by impairing their migration [25] and differentiation, ultimately compromising normal muscle development [13,25].

In addition, MO can induce H19 expression independent of ICR. MO creates a metabolically stressed intrauterine environment characterized by reduced oxygen availability and impaired placental oxygen transport. This condition can lead to fetal hypoxia, which activates hypoxia-responsive signaling pathways, particularly hypoxia-inducible factor 1α (HIF-1α) [13]. HIF-1α acts as a key transcriptional regulator that enables cells to adapt to low oxygen conditions. Under hypoxic stress, HIF-1α directly promotes the transcription of the long non-coding RNA H19 by binding to specific hypoxia response elements (HREs) within the H19 promoter region [26], thereby enhancing H19 expression in fetal tissues [13]. Beyond hypoxia, MO also generates a chronic pro-inflammatory intrauterine environment characterized by elevated inflammatory cytokines and metabolic stress signals [27]. Inflammatory signaling pathways have been shown to stimulate H19 expression [28]. Importantly, the inflammatory response induced by MO disrupts the chromatin architecture of the H19/*Igf2* imprinted locus, including epigenetic modifications at ICR [13]. Furthermore, MO suppresses key cellular energy-sensing pathways that regulate metabolic balance and mitochondrial function, leading to impaired mitochondrial biogenesis and altered mitochondrial activity in fetal skeletal muscle [10]. Mitochondrial metabolites such as α-ketoglutarate, is required for DNA demethylation and its deficiency may elevate DNA methylation of key metabolic regulatory loci [29], including peroxisome proliferator-activated receptor γ coactivator-1α (PGC-1α) [10] and possibly ICR and the promoter of H19.

## 3. H19 regulatory functions

H19 is a key regulator of embryonic development, postnatal growth, and metabolic homeostasis [3,13]. It modulates gene expression by recruiting chromatin-modifying enzymes, acting as a microRNA precursor and molecular sponge, and stabilizing regulatory proteins [13]. Through these mechanisms, H19 integrates environmental and cellular signals to regulate cell fate, tissue remodeling, and metabolic adaptation.

### 3.1. Epigenetic regulation by H19

**Fig. 3.**
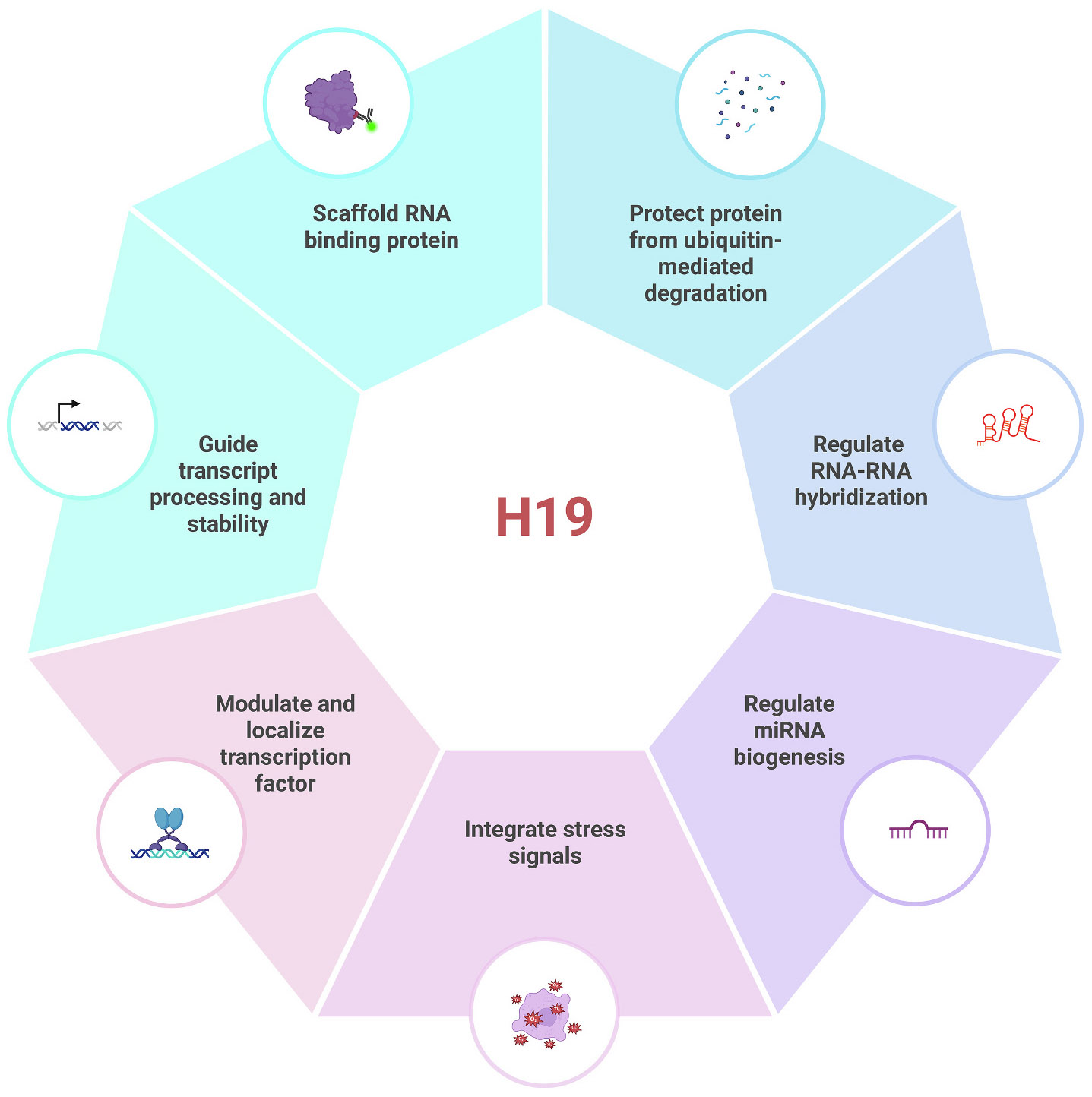
Multifunctional roles of lncRNA H19. Schematic illustrating the diverse post-transcriptional and cellular functions of H19.

Beyond the local control of *Igf2*, H19 also regulates broader chromatin dynamics. It interacts with methyl-CpG-binding domain protein 1 (MBD1) to form a repressive complex that recruits histone-modifying enzymes, including histone lysine methyltransferases. This complex establishes repressive histone marks such as H3K9me3 or H3K27me3 at promoters of growth-related imprinted genes like *Igf2*, *Mest*, and *Slc38a4*, leading to their silencing [30,31]. Through these ribonucleoprotein complexes, H19 acts as an epigenetic scaffold, coordinating DNA methylation and histone modification to fine-tune developmental gene expression (Fig. 2). In addition to these methylation-dependent mechanisms, H19 exerts epigenetic control through histone deacetylase regulation. The H19/miR-675 axis alleviates HDAC4 and HDAC5 activity, leading to increased histone acetylation at target gene promoters and suppresssing transcription of differentiation markers [32] (Fig. 2). By simultaneously influencing DNA and histone methylation, H19 integrates multiple layers of epigenetic regulation to cell proliferation and differentiation [3] (Fig. 3). Dysregulation at any of these levels can result in impaired growth and development [33].

H19 also functions as a molecular scaffold that recruits and coordinates chromatin-modifying enzymes, thereby exerting a strong influence on gene expression [3]. Acting as a scaffold means that H19 provides a structural platform through which chromatin modifiers are brought into proximity with their target genomic regions. Specifically, H19 binds to enhancer zeste homolog 2 (EZH2), the catalytic subunit of the Polycomb Repressive Complex 2 (PRC2), and directs it to promoters of specific genes [3]. EZH2 then catalyzes trimethylation of histone H3 at lysine 27 (H3K27me3), a histone mark associated with transcriptional repression [3]. Through this mechanism, H19 can promote the silencing of genes involved in differentiation. Overall, by acting as a scaffold for EZH2 and facilitating H3K27me3 deposition, H19 integrates epigenetic regulation into growth signaling networks [3]. Therefore, H19 acts as a key regulator of epigenetic homeostasis, controlling imprinting at its own locus, modulating chromatin states, and integrating environmental signals to shape developmental gene networks.

### 3.2. miRNA processing

H19 governs post-transcriptional control through a dual miRNA mechanism: it is both a precursor for embedded microRNAs and a competing endogenous RNA (ceRNA) that modulates the availability of numerous miRNAs. First, H19 is processed to generate miR-675-5p and miR-675-3p from exon 1 (Fig. 1). The maturation of miR-675 is context- and stress-responsive, tightly regulated by RNA-binding proteins such as HuR [33] and by the AKT- KHSRP (KH-type splicing regulatory protein) axis, which influences H19 cleavage efficiency and thereby tunes miR-675 output [13,33]. Functionally, miR-675 species restrain excessive proliferation and calibrate differentiation by targeting key growth and cell-cycle regulators (e.g., insulin-like growth factor 1 receptor (IGF1R), Smad factors, Cdc6 and by intersecting with canonical morphogen pathways [33,34]. Through these actions, the H19 → miR-675 provides a built-in “brake” that helps balance expansion with maturation during development [33,34].

Second, independent of its role as a precursor, H19 operates as a competing endogenous RNA (ceRNA) scaffold, sponging diverse miRNAs and thereby de-repressing their pro-growth, pro-differentiation, or pro-survival targets [35]. Across placenta [36], muscle [34], adipose tissue [37], bone [38], and neuronal systems [39], these miRNAs modulate IGF [35], TGF-β/SMAD [40], Wnt/β-catenin [41], PI3K-AKT [13], and mitogen-activated protein kinas (MAPK) signaling, particularly the p38 pathway [42], influencing cell differentiation, growth, and metabolic homeostasis. Notably, miR-675 [13,33,34,40] and let-7 [35,43,44] emerge as central regulators across multiple tissues, highlighting the broad impact of H19-mediated post-transcriptional regulation in development and disease (Table 1).

Mechanistically, the two arms reinforce each other: (i) by producing miR-675, H19 imposes targeted repression on proliferation drivers and certain pathway nodes, (ii) by sponging other miRNAs, H19 de-represses complementary sets of transcripts that promote orderly differentiation, matrix production, metabolism, and stress tolerance (Table 1).

### 3.3. Protein stabilization & RNA–protein interactions

Beyond its roles in epigenetic control and miRNA processing, H19 exerts a critical influence on protein stability and RNA–protein interactions, ensuring that key regulatory factors remain active and protected from premature degradation. This function is central to maintaining proteostasis, coordinating stress responses, and fine-tuning developmental programs [56]. One major mechanism involves H19 acting as a scaffold for RNA-binding proteins (RBPs) [57,58]. By directly associating with proteins such as HuR, KHSRP, and TDP43 (TAR DNA-binding protein 43), H19 regulates the processing, stability, and translational fate of target transcripts [35,57,58]. These complexes often form in response to developmental cues or stress signals, enabling precise control of gene expression (Fig. 3).

H19 can stabilize proteins by preventing ubiquitin-mediated degradation, binding to target proteins and protecting them from E3 ligase activity to maintain their function [59,60]. In addition, H19 modulates lipid and metabolic regulation by interacting with RNA-binding proteins such as PTBP1 to influence the activity and localization of transcription factors like SREBP1, thereby shaping transcriptional programs that control lipid metabolism and energy balance [61,62] (Fig. 2). Furthermore, H19 can recruit and stabilize transcription factors, such as FOXC2, enhancing their promoter binding and activation of downstream developmental pathways, thereby reinforcing lineage-specific gene expression programs [63].

### 3.4. Signaling pathway regulation

H19 exerts important control over TGF-β signaling, both canonical (Smad-dependent) and non-canonical. TGF-β signaling plays a crucial role in regulating cell proliferation, differentiation, and extracellular matrix remodeling, thereby controlling tissue development, repair, and fibrosis [32,44]. By interacting with SMAD regulators, sponging inhibitory miRNAs, or modulating histone deacetylases, H19 shapes the output of TGF-β pathways [32]. Through this modulation, H19 ensures balanced tissue remodeling and prevents pathological activation of TGF-β cascades.

A particularly well-documented regulatory node is the Wnt/β-catenin pathway, a master driver of developmental fate decisions. H19 reinforces Wnt/β-catenin activity by sponging regulators such as miR-625-5p [50], or miR-541-3p [64], while also interacting with transcriptional cofactors that promote Wnt target gene activation [50]. Inhibition of this pathway consistently abolishes H19’s pro-developmental effects, confirming Wnt/β-catenin as a central downstream effector of H19’s regulatory network [64].

H19 also intersects with p53, which together balance proliferation, apoptosis, and differentiation. By modulating p53 transcriptional activity, H19 prevents premature apoptosis while simultaneously guiding lineage specification [65]. Additionally, H19 promotes lipid droplet degradation in hepatic stellate cells by activating the LKB1-AMPK pathway, enhancing AMPK phosphorylation, lipid oxidation, and subsequent HSC activation [66]. Separately, H19 exerts neuroprotective effects by up-regulating VEGF signaling, leading to reduced neuronal apoptosis and preservation of cognitive function [55].

By engaging at multiple levels – epigenetic, post-transcriptional, and protein-stabilizing - H19 adjusts the intensity and duration of these signals, ensuring coordinated developmental progression (Fig. 3).

## 4. H19 in regulating organ and tissue development

### 4.1. Role of H19 in placenta and fetal development

H19 expression in the placenta is tightly regulated and shows a bimodal increase during cytotrophoblast differentiation, with two distinct expression peaks corresponding to early and late stages of maturation, independent of cell fusion or multinucleation [67]. Interestingly, H19 shows transient biallelic expression during early placental development before switching to maternal monoallelic expression after ~10 weeks of gestation, suggesting an imprinting-independent role in promoting early placental growth that is distinct from *Igf2* regulation [68].

On the other hand, H19 assumes a growth-restrictive role during late gestation, preventing uncontrolled placental expansion. Its loss results in persistent trophoblast proliferation and abnormal placental growth, highlighting its role as a physiological growth suppressor [33]. In the situation of fetal growth restriction (FGR), H19 suppresses trophoblast cell proliferation. Accordingly, abnormally reduced H19 expression in the placenta may contribute to excessive trophoblast proliferation [36,69]. Similar downregulation has also been documented in placentas from small-for-gestational-age (SGA) infants, even in the absence of overt structural abnormalities [70]. However, other studies have noted elevated H19 expression in FGR, possibly reflecting compensatory upregulation or temporal and context-specific feedback mechanisms [69]. These findings suggest that both reduced and excessive H19 expression can be detrimental, depending on developmental stage and disease context.

Assisted reproductive technologies (ART), such as IVF and intracytoplasmic sperm injection (ICSI), highlight the sensitivity of H19 expression to environmental conditions. ART pregnancies often show increased H19 and reduced *Igf2* expression in placental tissue [71]. Dysregulation of the H19/miR-675 axis may impair placental development by limiting IGF–PI3K–AKT–mTOR signaling, potentially leading to subtle developmental consequences [72].

H19 is also implicated in preeclampsia and intrauterine growth restriction, where reduced H19 and miR-675 levels are linked to abnormal trophoblast proliferation and impaired placental development [36,73]. In intrauterine growth restriction (IUGR), alterations in H19/*Igf2* imprinting are observed, with reduced methylation at the 11p15.5 ICR in normotensive IUGR placentas, suggesting distinct mechanisms compared to preeclampsia-associated IUGR [74].

### 4.2. Role of H19 in myogenesis and muscle development

#### 4.2.1. Muscle growth and development

Skeletal muscle accounts for approximately 30–50% of adult body mass and serves as a major site for glucose and fatty acid utilization [75]. It is also the primary tissue implicated in insulin resistance observed in obesity and T2D [76–78]. Skeletal myogenesis is a tightly regulated process that involves the differentiation of mesodermal cells into myogenic precursor cells, myoblasts, and eventually myotubes. This progression is controlled by the sequential expression of key markers, including *Pax7* (a marker for myogenic precursor cells) and a group of myogenic regulatory factors such as *Myf5*, *Myod*, *Myogenin*, and *Mrf4* [13,79]. The first phase of myogenesis takes place during the embryonic stage, leading to the formation of primary myofibers. A second wave of myogenesis occurs around mid-gestation in fetuses, during which the majority of muscle fibers are formed [79].

While H19 is typically repressed postnatally in many tissues, it remains highly expressed in skeletal muscle, signifying its continued functional relevance in muscle physiology [13]. H19 operates as a molecular sponge for various microRNAs, such as miR-935 and miR-296-5p, thereby lifting repression on their respective pro-myogenic targets and modulating gene expression networks essential for muscle proliferation and differentiation [42] (Table 1). It inhibits muscle growth by suppressing *Igf2*, a potent growth factor, and enhancing myostatin, an inhibitor of muscle hypertrophy [31]. Deletion of H19 leads to increased muscle mass through both hypertrophy and hyperplasia [31]. H19 suppresses muscle development by negatively regulating *Myog* mRNA stability in differentiating myoblasts, consistent with its inhibitory role in *Myog* expression in undifferentiated myoblasts [56]. Loss of H19 has also been associated with increased expression of myogenic differentiation markers, including MYOG and MYHC isoforms (Myosin Heavy Chain), resulting in improved myotube formation and accelerated muscle regeneration [31]. H19 negatively regulates muscle development by reducing miR-675 biogenesis and inhibiting IGF/AKT/KHSRP signaling [13]. Additionally, in aging muscle, H19 and its derived microRNAs miR-675-3p/5p are elevated and are negatively associated with anabolic SMAD1/5 expression, which correlates with reduced muscle fiber size and lower lean muscle mass (sarcopenia) in older adults [40]. These findings suggest that H19 is a negative regulator of skeletal muscle development.

MO impairs fetal skeletal muscle development by disrupting the bioconversion of lncRNA H19 to its embedded microRNA, miR-675. H19 inhibits, but miR-675 promotes myogenesis [13]. MO increases H19 expression, but reduces miR-675 biogenesis, leading to fewer differentiated myocytes and diminished expression of key myogenic genes, including *Myf5*, *Myod1*, and *Myog*, showing the bioconversion of H19 to miR-675 is a key regulatory factor [13]. MO disrupts myogenesis by altering the H19/*Igf2* ICR through hypermethylation, resulting in biallelic expression of H19 [13,25]. Additionally, MO suppresses *Igf2* expression by upregulating H19 during the neonatal stage, which subsequently contributes to impaired muscle development, metabolic changes, and functional deficits in adulthood [3].

H19 also plays a key role in maintaining skeletal muscle insulin sensitivity, and its dysregulation contributes to diabetes development. It is significantly downregulated in skeletal muscle of individuals with type 2 diabetes and insulin-resistant rodents, resulting in increased let-7 activity, impaired insulin signaling, and reduced glucose uptake, thereby establishing a double-negative feedback loop between H19 and let-7 that promotes metabolic dysfunction [80]. Beyond this ceRNA-mediated mechanism, muscle-enriched H19 further enhances insulin sensitivity by activating AMPK, at least in part through the downstream effector DUSP27, whereas its reduction in insulin-resistant states compromises glucose metabolism [81]. In parallel, diminished H19 expression in diabetic and high-fat diet conditions elevates HDAC6 activity and suppresses insulin receptor substrate 1 (IRS1) and contributes to insulin resistance [82].

#### 4.2.2. Myogenic migration

Myogenic cell migration is a critical step in embryonic muscle development, enabling the formation of limb muscles. Myogenic progenitor cells originate from the ventrolateral lip of the dermomyotome and undergo epithelial-to-mesenchymal transition to acquire migratory capacity. This process is tightly regulated by spatial and temporal gene expression and signaling pathways and occurs independently of muscle differentiation [25].

H19 plays a regulatory role in this migratory process [33]. MO elevates expression of H19, which disrupts embryonic myogenic cell migration [25]. Effective migration requires the formation of focal adhesions through proteins such as integrins [83], focal adhesion kinase (FAK) [84], paxillin (PXN), and talin (TLN) [25], which link the extracellular matrix (ECM) to the actin cytoskeleton and allow cells to move directionally. In MO limbs, key migratory genes such as *Pxn* and *Tln2* are downregulated, alongside reduced ECM organization, indicating impaired cell migration capacity [25]. Furthermore, MO suppresses signaling pathways critical for guiding migration, including hepatocyte growth factor, Wnt11, and fibroblast growth factor pathways [25]. The combined effect of elevated H19 and suppressed migratory and signaling gene expression likely creates a detrimental environment for embryonic muscle development.

#### 4.2.3. Muscle regeneration

H19 plays a complex role in muscle regeneration. Following skeletal muscle injury, regeneration begins with a regulated inflammatory phase involving infiltration of immune cells such as macrophages, eosinophils, regulatory T cells (Tregs), and neutrophils, along with cytokine release [85]. Fibroadipogenic progenitors (FAPs), located between muscle fibers, support muscle regeneration by promoting satellite cell proliferation. Satellite cells, marked by the transcription factor Pax7 in their quiescent state [86], are activated by injury signals and proliferate and fuse into multinucleated myotubes to form new muscle fibers [85]. H19 deficiency enhanced regenerative capacity following cardiotoxin-induced injury, which was accompanied by the upregulation of imprinted gene network and the IGF signaling pathway [31]. H19 contributes to muscle regeneration through its processed microRNAs, miR-675-3p and miR-675-5p, which promote myogenesis by downregulating Smad transcription factors and Cdc6, known inhibitors of muscle development [34]. Conversely, in chronic conditions such as COPD (Chronic Obstructive Pulmonary Disease), elevated H19 and miR- 675 levels are linked to reduced myoblast proliferation and impaired muscle regeneration, likely due to epigenetic alterations at the H19 locus [87].

### 4.3. Role of H19 in osteogenesis

#### 4.3.1. Osteogenic differentiation

H19 plays a vital regulatory role in osteogenic differentiation. H19 acts as a positive regulator of osteogenic differentiation in human adipose-derived stem cells (hASCs) and mesenchymal stem cells (MSCs) during the process by enhancing the expression of ALP, OCN, RUNX2, OSX and BMP markers. On the other hand, the knockdown of H19 impairs osteogenesis via H19/miR-675/HDAC axis [41,47].

Notably, H19 exhibits a dosage- and stage-dependent (biphasic) role during osteogenic differentiation. Low or transient H19 expression promotes osteogenesis, whereas sustained or excessive expression inhibits differentiation. This tightly regulated pattern is evident in BMP9-induced osteogenesis, where H19 is transiently upregulated during early differentiation to stimulate osteogenic gene expression, while both persistent overexpression and silencing impair BMP9-mediated osteogenesis [88,89]. Together, these findings indicate the requirement for precise temporal control of H19 expression during osteogenesis.

In addition, H19 regulates osteogenic differentiation by functioning as a competing endogenous RNA and microRNA host, thereby relieving repression of osteogenic transcription factors and signaling pathways (Table 1). Through interactions with microRNAs such as miR-140-5p [53] and miR-19b-3p [48], H19 enhances osteogenic gene expression and modulates the balance between osteogenesis and adipogenesis. In addition, H19 activates pro-osteogenic signaling pathways, including Wnt/β-catenin signaling, to drive osteoblast differentiation [41] (Table 1).

#### 4.3.2. Bone regeneration and repair

Beyond differentiation, H19 plays an essential role in bone regeneration and repair. H19 expression is significantly upregulated during fracture healing, particularly at days 4, 8, and 12 post-injury, where it promotes osteoblast and chondrocyte proliferation and reduces apoptosis, thereby supporting effective bone repair [90]. Consistent with this regenerative role, H19 overexpression enhances bone formation and homeostasis, whereas its dysregulation impairs repair processes [91].

H19 also contributes to bone regeneration under pathological and hormonal conditions. In ovariectomized (OVX) mice and postmenopausal osteoporosis (PMOP) patients, H19 expression is reduced, correlating with impaired osteogenesis and increased marrow adiposity. Restoration of H19 expression enhances bone formation and suppresses adipogenic differentiation, highlighting its therapeutic potential [63,64]. Estrogen deficiency decreases H19 levels while increasing inhibitory microRNAs, whereas estrogen replacement rescues H19 expression and osteogenic capacity, thereby mitigating bone loss [51].

Additionally, H19 regulates bone regeneration by shaping the local microenvironment, as exosomal H19 from bone marrow–derived MSCs enhances osteogenesis and angiogenesis, improving bone microstructure and vascularization [92]. Mechanical loading further induces H19 expression in MSCs, and loss of H19 impairs load-induced osteogenesis, underscoring its role in mechanotransduction during bone repair [38].

### 4.4. Role of H19 in neurogenesis

The central nervous system (CNS), comprising primarily neurons and glial cells, is a highly complex structure. H19 is highly expressed in tissues derived from the mesoderm, endoderm, neuroectoderm, and neural crest [93]. Moreover, genomic imprinting - most prominent in the placenta and brain - plays a key role in regulating neurogenesis and neural stem cell differentiation during brain development and maturation [94].

H19 plays a regulatory role in neural stem cell differentiation, with its expression influencing the balance between stemness and neuronal commitment. In induced neural stem cells (iNSCs), H19 expression is induced, and H19 silencing promotes neural differentiation without inducing apoptosis [39]. These findings reveal that reduced H19 levels favor neuronal lineage commitment during early differentiation stages.

In contrast to its role in inhibiting differentiation, H19 is essential for maintaining neural stem/progenitor cell (NSPC) proliferation and survival, particularly under injury-related stress conditions. During oxygen–glucose deprivation/reperfusion (OGD/R), H19 knockdown markedly suppresses NSPC proliferation, induces G0/G1 cell cycle arrest, and increases apoptosis [65]. This is accompanied by reduced expression of proliferation and early neuronal markers (Ki-67 and DCX) and increased levels of pro-apoptotic proteins, including p53, BAX, and cleaved caspase-3, alongside decreased BCL-2 expression. Conversely, H19 overexpression enhances NSPC proliferation and differentiation and protects against OGD/R-induced apoptosis [65].

H19 regulates neurogenesis during brain injury in a context-dependent manner, where its upregulation promotes neural stem cell proliferation and reduces apoptosis, supporting regeneration [95]. However, excessive or sustained H19 expression can impair neurogenesis, as elevated circulating H19 correlates with increased stroke severity, while H19 knockdown reduces brain damage and improves neurological recovery [96].

### 4.5. Role of H19 in adrenal gland and steroidogenesis

H19 is robustly expressed in normal adult adrenal tissue and in benign adrenocortical tumors and is positively regulated by adrenocorticotropic hormone (ACTH), whereas its pronounced downregulation accompanied by elevated *Igf2* expression in hormonally active adrenocortical carcinomas suggests that loss of H19 may contribute to adrenal tumorigenesis [97]. High cortisol levels activate glucocorticoid receptor (GR) signaling, which increases DNA methyltransferases DNMT3a/3b expression and enhances H19 methylation, leading to reduced H19 expression, elevated let-7 activity, decreased steroidogenic acute regulatory protein (StAR) expression, and ultimately impaired adrenal steroid synthesis in fetal tissues. These effects persist across multiple generations through maternal inheritance [98].

Precise regulation of steroid hormone production is critical for reproductive function, as it governs key processes such as follicular maturation, ovulation, and coordination of endometrial receptivity [99]. H19 functions as a competing endogenous RNA that sequesters microRNA let-7, thereby relieving let-7-mediated repression of (StAR) at the post-transcriptional level. Overexpression of H19 enhances StAR expression in human and murine cell models [99]. Loss of H19 reduces ovarian Cyp17 expression and serum testosterone in mice, whereas elevated H19 levels are observed in women with PCOS and are associated with increased androgen production [100].

These findings reveal that H19 is a critical epigenetic regulator of adrenal and ovarian steroidogenesis whose dysregulation contributes to endocrine disorders, including adrenal tumorigenesis and PCOS-associated hyperandrogenemia, and may link developmental programming to long-term reproductive and metabolic disease.

## 5. Conclusion and future perspective

Maternal obesity, gestational diabetes, and other metabolic conditions initiate a cascade of lasting health issues in offspring, primarily through epigenetic and metabolic programming. Central to this process is H19, a crucial regulator of development. H19 operates through several key mechanisms, including controlling genomic imprinting at the H19/*Igf2* locus, acting as a molecular sponge for microRNAs, and modulating the stability of key signaling proteins. When MO disrupts the delicate balance of the H19/ *Igf2* axis, it directly impairs fetal growth and development, thereby increasing the offspring’s vulnerability to chronic conditions like metabolic disorders. Of note, this review is limited by the availability of organ-specific and mechanistic studies in certain tissues, and further comprehensive investigations are needed to fully elucidate the systemic roles of H19 in developmental programming. Future studies may leverage advanced techniques like single-cell and spatial transcriptomics to precisely map how H19’s epigenetic regulation is altered across different tissues and cell types, which alter fetal development and potentially pass down through generations. Due to its potent regulatory role, H19 and its downstream mediators hold significant promise as both biomarkers for risk assessment and therapeutic targets for interventions aimed at mitigating inherited metabolic risk, offering a pathway to disrupt the cycle of obesity-related health issues.

## Figures and Tables

**Table 1:** Tissue-specific roles of H19 in microRNA processing and signaling regulation.

miRNA	H19 Role	Target Pathway	Outcome	Ref.
**Placenta**				
miR-675	Host/precursor (H19 → miR-675)	Targeting IGF1R and reducing its expression	Restricting placental growth before birth	[33]
		Downregulation of Nodal Modulator 1 (NOMO1)	Reducing the proliferation of human trophoblast cells.	[36]
miR-18a-5p	Endogenous sponge; Negative correlation (↓ H19 → ↑ miR-18a-5p)	Downregulation of interferon regulatory factor 2 (IRF2)	Inhibiting two signaling pathways: PI3K/AKT/mTOR and MAPK/ERK/mTOR pathway; Reducing viability and growth of extravillous trophoblast cells	[45]
miR-19a/b	Sponges miR-19a/miR-19b; (↑ H19 → ↓ miR-19a/b)	Increasing SMAD4	Suppressing placental cell proliferation and growth	[46]
**Muscle**				
miR-675	Host/precursor (H19 → miR-675)	Suppressing SMAD1/5 pathway	Reducing muscle mass in older individuals	[40]
	Under MO condition (↑ H19 → ↓ miR-675)	Blocking phosphorylation of KHSRP	Suppressing IGF2/AKT signaling and myotube formation	[13]
let-7	Sponges let-7 (↓ H19 → ↑ let-7)	Downregulating HMGA2 (High Mobility Group AT-hook 2) and DICER (Dicer ribonuclease)	Enhancing muscle differentiation	[35]
miR-935/ miR-296-5p	H19 ↑ → miR-935/miR-296-5p ↓	Upregulating DBN (drebrin 1) expression and activating p38 Mitogen-Activated Protein Kinase (MAPK) signaling pathway	Decreasing the proliferation of skeletal muscle satellite cells	[42]
**Bone**				
miR-149	H19 ↑ → miR-149 ↓	Upregulating stromal cell-derived factor 1 (SDF-1)	Increasing alkaline phosphatase (ALP) activity, osteocalcin (OCN) content, calcium deposition, and upregulating the expression of key osteogenic proteins, including ALP, OCN, like Runt-related transcription factor 2 (RUNX2), and osterix (OSX)	[47]
miR-22 miR-141	H19 ↑ → miR-22/miR-141↓	Inhibiting Wnt/β-catenin pathway	Reducing osteogenic markers CD29, CD90, CD44, CD57, Nestin and Sox10	[41]
miR-138	H19 ↑ → miR-138 ↓	Targeting protein tyrosine kinase 2 (PTK2), a gene encoding focal adhesion kinase (FAK), and upregulating FAK expression	Activating downstream osteogenic pathways, such as FAK-ERK1/2-Runx2 signaling	[38]
the miR-19b-3p	H19 ↓ → miR-19b-3p ↑	Promoting alkaline phosphatase (ALP) activity	Enhancing key osteogenic markers RUNX2 and collagen type I alpha 1 chain (COL1A1)	[48]
miR-675	Host/precursor (H19 → miR-675)	Inhibiting Adenomatous Polyposis Coli (APC), thereby stabilizing β-catenin, enhancing its nuclear translocation, and activating Wnt/β-catenin.	Upregulating early and late osteogenic markers, including ALP, RUNX2, OCN, and OSX	[49,50]
miR-532-3p	H19 ↓ → miR-532-3p ↑	Suppressing sirtuin 1 (SIRT1)	Negatively regulating osteogenic differentiation	[51]
miR-185-5p	H19 ↑ → miR-185-5p ↓	Increasing insulin-like growth factor 1 (IGF1)	Enhancing osteogenic marker expression and mineralization	[52]
miR-140-5p	H19 ↑ → miR-140-5p ↓	Increasing special AT-rich sequence-binding protein 2 (SATB2)	Promoting key osteogenic markers, such as Runx2 and ALP	[53]
miR-29b-3p	H19 ↑ → miR-29b-3p ↓	Increasing Dickkopf-related protein 1 (DKK1), thereby suppressing Wnt signaling	Reducing expression of osteogenic markers like Runx2, OCN, and osteopontin (OPN).	[54]
**Neuron**				
miR-325-3p	H19 ↑ → miR-325-3p ↓	Upregulating C-terminal binding protein 2 (CTBP2)	Suppressing neural differentiation	[39]
miR-107	H19 ↑ → miR-107↓	Enhancing vascular endothelial growth factor (VEGF) expression,	Inhibiting neuronal apoptosis and preserving cognitive function	[55]

## Data Availability

No data was used for the research described in the article.
